# Single low-dose primaquine for blocking transmission of *Plasmodium falciparum* malaria – a proposed model-derived age-based regimen for sub-Saharan Africa

**DOI:** 10.1186/s12916-017-0990-6

**Published:** 2018-01-18

**Authors:** W. Robert Taylor, Htee Khu Naw, Kathryn Maitland, Thomas N. Williams, Melissa Kapulu, Umberto D’Alessandro, James A. Berkley, Philip Bejon, Joseph Okebe, Jane Achan, Alfred Ngwa Amambua, Muna Affara, Davis Nwakanma, Jean-Pierre van Geertruyden, Muhindo Mavoko, Pascal Lutumba, Junior Matangila, Philipe Brasseur, Patrice Piola, Rindra Randremanana, Estrella Lasry, Caterina Fanello, Marie Onyamboko, Birgit Schramm, Zolia Yah, Joel Jones, Rick M. Fairhurst, Mahamadou Diakite, Grace Malenga, Malcolm Molyneux, Claude Rwagacondo, Charles Obonyo, Endalamaw Gadisa, Abraham Aseffa, Mores Loolpapit, Marie-Claire Henry, Grant Dorsey, Chandy John, Sodiomon B. Sirima, Karen I. Barnes, Peter Kremsner, Nicholas P. Day, Nicholas J. White, Mavuto Mukaka

**Affiliations:** 1 Mahidol Oxford Tropical Medicine Research Unit (MORU), Mahidol University, 420/6 Rajvithi Road, Rajthevee, Bangkok, 10400 Thailand; 2 Centre for Tropical Medicine and Global Health, Nuffield Department of Medicine, University of Oxford, Oxford, UK; 3 Division of Tropical and Humanitarian Medicine, University Hospitals of Geneva, Geneva, Switzerland; 4 KEMRI–Wellcome Trust Research Programme, Centre for Geographic Medicine Research-Coast, Kilifi, Kenya; 5 Wellcome Trust Centre for Clinical Tropical Medicine and Department of Paediatrics, Faculty of Medicine, Imperial College, London, UK; 6 MRC Unit, Fajara, Banjul, The Gambia; 7 Faculty of Infectious and Tropical Diseases, London School of Hygiene and Tropical Medicine, London, UK; 8 Global Health Institute, University of Antwerp, Antwerp, Belgium; 9 Department of Tropical Medicine, University of Kinshasa, Kinshasa, Democratic Republic of Congo; 10 Unité Mixte de Recherche 198, URMITE, IRD, Dakar, Sénégal; 11 Institut Pasteur de Madagascar, BP 1274 Antananarivo, Madagascar; 12 Médecins Sans Frontières, Paris, France; 13 Kinshasa Mahidol Oxford Research Unit, Kinshasa, Democratic Republic of Congo; 14 Kinshasa School of Public Health, Kinshasa, Democratic Republic of Congo; 15 Epicentre, Paris, France; 16 National Malaria Control Programme, Monrovia, Sierra Leone; 17 Laboratory of Malaria and Vector Research, National Institute of Allergy and Infectious Diseases, National Institutes of Health, Rockville, MD USA; 18 Malaria Research and Training Centre, USTTB, Bamako, Mali; 19 Queen Elizabeth Hospital, Blantyre, Malawi; 20 Malawi-Liverpool-Wellcome Trust Clinical Research Programme, Blantyre, Malawi; 21 National Malaria Control Programme, Kigali, Rwanda; 22 Kenya Medical Research Institute, Kisumu, Kenya; 23 Armauer Hansen Research Institute, Addis Ababa, Ethiopia; 24 Amref Health Africa in Kenya, Nairobi, Kenya; 25 Centre de Recherche Entomologique de Cotonou, Cotonou, Benin; 26 Department of Medicine, University of California San Francisco, San Francisco, CA USA; 27 Department of Pediatrics, Indiana University, Indianapolis, IN USA; 28 Centre National de Recherche et de Formation sur le Paludisme, Ouagadougou, Burkina Faso; 29 Groupe de Recherche Action en Santé (GRAS), Ouagadougou, Burkina Faso; 30 Division of Clinical Pharmacology, Department of Medicine, University of Cape Town, Cape Town, South Africa; 31 Institute of Tropical Medicine, University of Tubingen, Tubingen, Germany

**Keywords:** Primaquine, Age-based dosing, *Plasmodium falciparum*, Malaria, Transmission blocking

## Abstract

**Background:**

In 2012, the World Health Organization recommended blocking the transmission of *Plasmodium falciparum* with single low-dose primaquine (SLDPQ, target dose 0.25 mg base/kg body weight), without testing for glucose-6-phosphate dehydrogenase deficiency (G6PDd), when treating patients with uncomplicated falciparum malaria. We sought to develop an age-based SLDPQ regimen that would be suitable for sub-Saharan Africa.

**Methods:**

Using data on the anti-infectivity efficacy and tolerability of primaquine (PQ), the epidemiology of anaemia, and the risks of PQ-induced acute haemolytic anaemia (AHA) and clinically significant anaemia (CSA), we prospectively defined therapeutic-dose ranges of 0.15–0.4 mg PQ base/kg for children aged 1–5 years and 0.15–0.5 mg PQ base/kg for individuals aged ≥6 years (therapeutic indices 2.7 and 3.3, respectively). We chose 1.25 mg PQ base for infants aged 6–11 months because they have the highest rate of baseline anaemia and the highest risks of AHA and CSA. We modelled an anthropometric database of 661,979 African individuals aged ≥6 months (549,127 healthy individuals, 28,466 malaria patients and 84,386 individuals with other infections/illnesses) by the Box–Cox transformation power exponential and tested PQ doses of 1–15 mg base, selecting dosing groups based on calculated mg/kg PQ doses.

**Results:**

From the Box–Cox transformation power exponential model, five age categories were selected: (i) 6–11 months (*n* = 39,886, 6.03%), (ii) 1–5 years (*n* = 261,036, 45.46%), (iii) 6–9 years (*n* = 20,770, 3.14%), (iv) 10–14 years (*n* = 12,155, 1.84%) and (v) ≥15 years (*n* = 328,132, 49.57%) to receive 1.25, 2.5, 5, 7.5 and 15 mg PQ base for corresponding median (1st and 99th centiles) mg/kg PQ base of: (i) 0.16 (0.12–0.25), (ii) 0.21 (0.13–0.37), (iii) 0.25 (0.16–0.38), (iv) 0.26 (0.15–0.38) and (v) 0.27 (0.17–0.40). The proportions of individuals predicted to receive optimal therapeutic PQ doses were: 73.2 (29,180/39,886), 93.7 (244,537/261,036), 99.6 (20,690/20,770), 99.4 (12,086/12,155) and 99.8% (327,620/328,132), respectively.

**Conclusions:**

We plan to test the safety of this age-based dosing regimen in a large randomised placebo-controlled trial (ISRCTN11594437) of uncomplicated falciparum malaria in G6PDd African children aged 0.5 − 11 years. If the regimen is safe and demonstrates adequate pharmacokinetics, it should be used to support malaria elimination.

**Electronic supplementary material:**

The online version of this article (doi:10.1186/s12916-017-0990-6) contains supplementary material, which is available to authorized users.

## Background

In an effort to stem the continuing emergence of artemisinin-resistant *Plasmodium falciparum* (ARPf) from the Greater Mekong Subregion to other parts of the world [1–8], the World Health Organization (WHO) has recommended the addition of single low-dose primaquine (SLDPQ) to artemisinin-based combination therapy (ACTs) for treating acute uncomplicated falciparum malaria in low-transmission areas, particularly where ARPf is prevalent [9, 10]. The recommended target dose of 0.25 mg/kg body weight of PQ base was considered safe, even in patients with severe forms of glucose-6-phosphate dehydrogenase deficiency (G6PDd). Accordingly, the WHO recommended its adoption without G6PDd testing.

While SLDPQ is not currently deployed anywhere in sub-Saharan African (SSA), several low-endemicity countries, including Botswana, Eritrea, Swaziland and Zimbabwe (R. Cibulski, personal communication) have added SLDPQ to their national treatment guidelines and intend to go forward with SLDPQ treatment. The main reasons cited by ministries of health for not enacting the WHO recommendation is the fear that SLDPQ would cause acute haemolytic anaemia (AHA) in G6PDd individuals and the impossibility of widely deploying a suitable G6PDd test to exclude such individuals from receiving SLDPQ [11].

Despite this, there is an increasing body of evidence that SLDPQ is tolerated well by both malaria patients and healthy individuals with good pre-treatment haemoglobin (Hb) concentrations in both Southeast Asia [12] and SSA [13] (SAFEPRIM I and II, and PQSAFETY studies; ClinicalTrials.gov identifiers NCT02174900, NCT02654730, and NCT02535767). Moreover, pilot work has demonstrated the feasibility of deploying SLDPQ in Swaziland’s health system [14].

Important hurdles to using SLDPQ include the lack both of user-friendly age- or weight-based regimens and of suitable paediatric formulations and tablet strengths that have been produced to good manufacturing practice standards and registered to international standards. Although the WHO did recommend a weight-based dose regimen in 2015, it relies on tablet fractions [15].

We recently designed an age-based dosing regimen for Cambodia (and by extension several neighbouring countries) using four tablet strengths: 2.5, 5, 7.5 and 15 mg [16]. This regimen resulted in high proportions of patients who would receive an optimised transmission-blocking PQ dose, defined as 0.15–0.38 mg/kg body weight of PQ base, taking into account that the severe G6PDd Viangchan variant is prevalent in Cambodia [17, 18].

Age-based dosing regimens exist for several antimalarial drugs, including artesunate (AS)-mefloquine and AS-amodiaquine (AQ; ASAQ) [19]. They offer the substantial advantage of not requiring weighing scales and are suitable for the rapid distribution of drugs to large numbers of individuals, even under logistically challenging circumstances, for example, the mass drug administration (MDA) of ASAQ in Liberia during a recent Ebola outbreak [20]. Moreover, several SSA countries have adopted home-based management of malaria [21] and many patients buy their drugs from the informal private sector, where reliable weighing scales may not be available [22–25].

The development of an age-based dosing regimen requires a thorough examination of efficacy, tolerability, pharmacokinetics (PK), PK–pharmacodynamic (PD; PKPD) relationships, regimen simplicity, and suitable tablet strength availability. These aspects have been investigated elsewhere [16], but a recent study of PQ PK in *P. falciparum*-infected children aged 2–14 years from Burkina Faso illustrates the independent effects of age, weight and cytochrome 2D6 metaboliser status on PQ and carboxyPQ exposures [26]. Younger children and children with a lower body weight had lower PQ and carboxyPQ exposures, and poor PQ metabolisers had increased PQ but reduced carboxyPQ exposures. In this paper, we review aspects of SLDPQ pertinent to SSA and discuss how we determined our proposed age-based dosing regimen for SLDPQ.

### Low-dose primaquine efficacy in reducing mosquito infectivity

Early work demonstrated a rapid reduction in mosquito infectivity (within 15 hours) when low doses (e.g. 10 mg) of the 8-aminoquinoline pamaquine/plasmoquine were combined with standard antimalarial treatments in falciparum malaria patients [27, 28]. Later work suggested that 15 mg of PQ had a similar effect on mosquito infectivity as 45 mg (0.75 mg/kg in a 60-kg adult) [29, 30].

The comprehensive effect of SLDPQ on mosquito infectivity has been demonstrated by Dicko et al., who studied symptomatic [12/81 (14.8%)] and asymptomatic *P. falciparum*-infected Malian adults and children with ≥2 gametocytes on a thick blood film [31]. Given with the first dose of dihydroartemisinin-piperaquine (DHAPP), the mg/kg dose of PQ base was 0 (control), 0.0625, 0.125, 0.25 and 0.5. Efficacy was assessed by direct mosquito membrane feeding assays.

The primary efficacy endpoint was the mean within-person percentage reduction in mosquito infectivity for a given PQ-dosing group on study day 2 (D2), i.e. 48 hours in patients infected ≥1 mosquito pre- and post-PQ treatment:

100% × (*N* infected individuals at D0 – *N* infected individuals at D2)/*N* infected individuals at D0.

The 0.125-mg/kg dose (7.5 mg in a 60-kg individual) achieved a mean 94.9% [95% confidence interval (CI) 84.6–100] reduction in infectivity (*p* = 0.045) whereas the 0.0625-mg/kg dose (3.75 mg in a 60-kg individual) achieved a non-significant 59.83% reduction in infectivity (95% CI 16.9–100, *p* = 0.18). Consistent results come from a dose-ranging study in DHAPP-dosed Cambodian and Vietnamese *P. falciparum*-infected adults. A significant anti-infectivity effect was seen with 3.75 mg of PQ base and a maximal blocking effect was seen with 7.5 mg of PQ base [9].

Dicko et al*.* reconfirmed the pre-treatment non-linear gametocyte density–infectivity relationship [32–34], but following treatment there was no relationship between gametocyte density and mosquito infectivity. Consistent with these observations are the findings that febrile adults with patent gametocytaemia are more infectious than those who are afebrile with subpatent gametocytaemia [35], and that asymptomatic *P. falciparum* carriers (QT-NASBA-positive gametocytaemia) have low baseline [36] and post-treatment [artemether-lumefantrine (AL) or DHAPP] infectivity whether or not they also received SLDPQ [36, 37].

Based on the patient mosquito infectivity data, we chose the same minimum 0.15 mg/kg dose of PQ base as we did in Cambodia [16].

### Primaquine tolerability and safety

PQ is a well-tolerated and very safe drug. The mortality associated with PQ has been estimated at only 1 in 621,428 (upper 95% CI 1 in 407,807) patients treated, most having been associated with repeated PQ dosing as anti-relapse therapy for vivax malaria [38]. The three principal side effects of PQ, all dose-dependent, are abdominal pain, methaemoglobinaemia and AHA.

#### Gastrointestinal side effects

Occurrences of abdominal pain and vomiting have been unremarkable in recent trials in asymptomatic *P. falciparum*-infected African children, dosed with PQ on D2. PQ-induced abdominal pain and early vomiting necessitating retreatment were not reported by Goncalves et al*.* [36] or Eziefula et al*.* [39], but the former re-dosed 5/360 patients with AL on D0 or D1. Okebe et al*.* re-dosed 1/344 children with DHAPP and the reported frequencies of abdominal pain in the DHAPP-alone vs. DHAPP + PQ arms (0.2, 0.4 and 0.75 mg/kg) were 12, 14, 15 and 9%, respectively [37].

Similarly, 14 (2.5%) of 564 children treated with sulphadoxine-pyrimethamine (SP), artesunate and PQ on D2 vomited but none had persistent vomiting following retreatment [40]. Dicko et al*.* reported overall rates of mild abdominal pain of 10% and mild vomiting of 1.3% across all arms but no one required repeat dosing [31] whilst acutely infected falciparum patients of all ages (median 10 years) from Tanzania had identical rates of abdominal pain and vomiting of 6.4% (7/110) and 5.5% (6/110), respectively, in the AL-alone and the AL + SLDPQ arms [13].

When ACTs are given alone, early ACT-induced vomiting in uncomplicated malaria patients occurs primarily in children aged <5 years and, within this age band, the youngest children are at the highest risk. Rates of early vomiting tend to be significantly less frequent on D1 compared to D0. DHAPP is associated with the highest D0 rate of early vomiting (7–10%) whilst AL and ASAQ have overlapping rates (<3–6%) [41–44]. One trial reported very low early vomiting rates in adults and children aged >5 years: 0.4% (2/496) vs. 0% (0/502) in ASAQ (mean age 17.3 years) and AL-treated patients (mean age 16.5 years), respectively [44]. The WHO recommendation is to give SLDPQ with the first dose of ACT. Whether this would increase the rates of early vomiting in younger children, who, therefore, might be better dosed on D1 due to better tolerability, is unknown.

#### Methaemoglobinaemia

While no published PQ studies from Africa have measured methaemoglobin (metHb) concentrations, they are being measured in an ongoing study (ClinicalTrials.gov identifier NCT02535767). Methaemoglobinaemia manifests clinically as a blue discoloration of the lips and tongue when metHb concentrations are approximately 20%. metHb levels are mildly raised in malaria and correlate positively with disease severity. Mean levels of 2%, 4.1% and 5.8% were reported in healthy controls, uncomplicated malaria patients and severe malaria patients with death or clinical sequelae, respectively [45]. Data from Cambodia show that 0.25 mg/kg of PQ (L. Desoley, unpublished) in *P. falciparum*-infected patients and 0.75 mg/kg in *P. vivax*-infected patients resulted in a maximum metHb concentration of 3.6% and 4.9% [46], respectively. Thus, methaemoglobinaemia is not considered a clinical concern in SLDPQ-treated African children.

#### Acute haemolytic anaemia and African variants of G6PD deficiency

The most-feared PQ side effect is dose-related AHA. Previous experience with AHA induced by chlorproguanil dapsone, resulting in the stopping of its development, is a cautionary lesson [47, 48]. The incidence of AHA is greater in individuals with lower G6PD enzyme activities, so that hemizygous males and homozygous females are more likely to suffer PQ-induced AHA than heterozygous females with intermediate G6PD enzyme activities [49, 50].

The G6PDd A- variant (G202A), which in one study from Kenya accounted for most of the variance in G6PD enzyme activity [51], is the most common G6PD variant in SSA [52], with reported allele frequencies of ~2% in The Gambia, ~17% in Kenya and ~21% in Nigeria [53–55]. Although referred to as a mild variant, enzyme activities of ≤1 U/g Hb are well documented [56–58]. Other mild A- variants, reported from West Africa are G6PDd Mexico City (C680A) and Betica-Selma (A968G) [59].

G6PD Santamaria (T542A), a severe G6PDd variant, is also present in West Africa [59] and has a residual enzyme activity of ~2–3% of wild-type G6PD [60, 61]. It had a reported allele frequency of 6.6% in 2379 Gambian children [62]. In Sudan, the frequencies in males of the severe G6PD Mediterranean (≤3.8%) and G6PD A- variants (≤2.1%) were low in two studies [63, 64].

### Primaquine research on transmission blocking in Africa

PQ research in Africa is limited. Studies have included G6PDd and G6PD normal healthy individuals, malaria patients and asymptomatic *P. falciparum* carriers – all with high baseline Hb concentrations, usually ≥8 g/dL. G6PD status has been assessed by the qualitative fluorescent spot test or a qualitative rapid diagnostic test, supplemented by genotypic analysis in some studies.

#### G6PDd and G6PD normal healthy individuals

One MDA study examined 0.75 mg/kg of PQ base, administered on D2, in Tanzanian children aged <12 years (Hb ≥ 8 g/dL) who also received a standard dose of SP [40]. On D7, genotypically determined homozygous or hemizygous G6PDd children had the greatest fall in mean Hb: -2.5 (95% CI -1.2 to -3.8) g/dL vs. -1.6 (-0.9 to -2.2) g/dL in heterozygous females, and vs. -0.5 (-0.4 to -0.6) g/dL in G6PD normal children. No PQ PK analysis was performed but the authors noted that children aged <5 years were at risk of a greater fall in Hb, independent of G6PD status, if they received higher mg/kg doses of PQ. Indeed, an examination of the PQ-dosing regimen used shows that many children received doses far greater than the 0.75 mg/kg target dose, based on anthropometric data of the local population (Table 1) and those of our modelled database (Table 2).

**Table 1:** The primaquine (PQ) regimen administered to Tanzanian children aged <12 years by Shekalaghe et al. [40] and the mg/kg they received based on anthropometric data of the local population

		Weight (kg)			PQ received (mg/kg body weight)*		
PQ dose (mg base)	Age (years)	25th	Median	75th	25th	Median	75th
7.5	1–2	9.5	11.0	11.6	0.65	0.68	0.79
15	3–4	11.5	12.9	14.5	1.03	1.16	1.30
15	5–7	14.8	16.7	19.0	0.79	0.90	1.01
22.5	8–11	20.5	23.0	27.1	0.83	0.98	1.10

*PQ* primaquine

* Corresponding mg base/kg doses of PQ are shown for the 25th, 50th (median) and 75th centiles

**Table 2:** Calculated mg/kg of primaquine (PQ) base that would be received by children aged <12 years using the PQ regimen of Shekalaghe et al. [40] based on anthropometric data of the modelled database

	Modelled weights (kg)										
Age band (years)	Minimum	1st	5th	10th	25th	Median	75th	90th	95th	99th	Maximum
1–2	5.5	6.5	7.4	7.9	8.9	10.0	11.5	12.8	13.6	15.2	18.2
3–4	7.8	9.2	10.4	11.1	12.4	13.8	15.3	16.8	17.9	19.9	23.9
5–7	10.0	11.5	13.0	14.0	15.2	17.0	19.2	21.0	22.8	26.0	32.1
8–11	14.7	16.6	18.4	19.5	21.5	24.0	27.4	31.0	33.2	38.0	46.0
	PQ dose (mg base/kg)										
PQ dose (mg base)	Minimum	1st	5th	10th	25th	Median	75th	90th	95th	99th	Maximum
7.5	0.41	0.49	0.55	0.59	0.65	0.75	0.84	0.95	1.01	1.15	1.37
15	0.63	0.75	0.84	0.89	0.98	1.09	1.21	1.35	1.44	1.63	1.92
15	0.47	0.58	0.66	0.71	0.78	0.88	0.99	1.07	1.15	1.30	1.50
22.5	0.49	0.59	0.68	0.73	0.82	0.94	1.05	1.15	1.22	1.36	1.53

*PQ* primaquine

#### G6PDd and/or G6PD normal malaria patients

The Tanzanian study of Mwaiswelo et al*.* [13] showed that predominantly older children (median age 10 years, range 1 − 84) whose mean Hb was ~11 g/dL tolerated SLDPQ (0.25 mg/kg) well when given on D0 with the first dose of AL. The G6PDd heterozygous females (*n* = 4) in the AL-alone arm had the greatest mean absolute decline in Hb of ~1.7 g/dL whereas the G6PDd hemizygous males (*n* = 9) and homozygous females (*n* = 5) in the AL + SLDPQ arm had the greatest fall in Hb, a mean of ~1.5 g/dL with an upper 95% CI of ~2.4 g/dL, representing fractional falls from baseline of 12.6 and 19%, respectively. The corresponding data for these patients in the AL-alone group are 0.35 and 1.1 g/dL, and 2.7 and 9.7%.

In G6PD normal patients aged 1–10 years (Hb ≥ 8 g/dL), AL alone or combined with PQ (0.1, 0.4 or 0.75 mg/kg on D2) resulted in mean falls in Hb on D3 (the nadir in this study) of ~ -0.5 to ~ -0.75 g/dL that were unrelated to PQ dose. Indeed, the mean changes in Hb on D2 before PQ was given were very similar and explain most of the decline in Hb change post-PQ treatment [39]. On further analysis, these authors reported a PQ dose effect in the mean absolute fall in Hb on D7 (not seen on D3 or D10) in the small number of genotypically determined heterozygous females (*n* = 14) who received 0.75 mg/kg of PQ compared to those who received placebo [65]. The difference in the 0.75 vs. placebo arm was ~ -0.67 g/dL (*p* = 0.044).

When *P. falciparum*-infected children (3–15 years) were dosed on D3 with 0.75 mg base/kg of PQ, four G6PDd hemizygous males showed an increasing trend of a higher decline (median ~ -22%) in the fractional Hb on D7 compared to the 39 G6PD normal and nine G6PDd heterozygous females, who had median Hb falls of ~ -5% [66].

#### G6PD normal P. falciparum carriers

Goncalves et al*.* recruited children aged 2 − 15 years (Hb ≥ 8 g/dL) and reported small changes in mean Hb concentrations on D3 and D7 using AL alone or AL plus 0.25 or 0.4 mg/kg of PQ on D2 [36]. The absolute mean maximal fall in Hb (measured at the time of maximum fall) was highest in the 0.4-mg/kg arm but this was significantly different (*p* = 0.006) only when compared to placebo: -1.21 (95% CI -1.45 to -0.97) vs. -0.71 (-0.98 to -0.44). The proportion of patients with Hb falls ≥2 g/dL were broadly similar (*p* = 0.5) between the AL alone [9/62 (14.5%)], and the AL plus 0.25-mg/kg [12/75 (16%)] or 0.4-mg/kg PQ [14/73 (19.2%)] arms.

Okebe et al*.* also reported low and transient mean declines in Hb concentrations at D7 in just over half of treated asymptomatic *P. falciparum* carriers (DHAPP alone and DHAPP plus three PQ doses). The highest mean decline in Hb occurred on D3 and most subjects recovered their Hb concentration by D10 [37].

#### Unpublished G6PDd healthy individuals and P. falciparum carriers – SAFEPRIM and PQSAFETY studies

Three studies have assessed the tolerability of SLDPQ in G6PDd males (determined by the fluorescent spot test) aged 18–45 years with asymptomatic *P. falciparum* infection (Burkina Faso, *n* = 50) or healthy volunteers aged ≥10 years (The Gambia, *n* = 50), who had baseline Hb concentrations ≥11 g/dL and were treated with either AL (Burkina Faso, SAFEPRIM I) or DHAPP (The Gambia, SAFEPRIM II). The PQ doses (0.25 and 0.4 mg/kg) used were tolerated well and the falls in Hb were generally small when assessed at D7 (U. D’Alessandro, personal communication). The Hb dynamics were characterised by an initial decline in mean Hb followed by recovery, a pattern similar to that in patients with acute uncomplicated falciparum malaria.

In a small PQ PK study (ClinicalTrials.gov identifier NCT02535767) of healthy G6PDd Malian adults with Hb concentrations ≥10 g/dL, PQ doses of 0.4, 0.45 and 0.5 mg/kg were also tolerated well (I. Chen, personal communication), with overlap of the fractional and absolute falls in Hb in the G6PD normal and G6PDd groups given 0.5 mg base/kg of SLDPQ.

#### Summary of PQ research in Africa

These studies, in which individuals had relatively high pre-treatment Hb concentrations, have provided useful data on Hb dynamics in patients of all ages as well as healthy G6PD normal and G6PDd *P. falciparum* carriers. The declines in Hb appeared well tolerated despite falls ≥2 g/dL in some patients. These data show that the declines in Hb in PQ-treated hemizygous males and homozygous females overlap with heterozygous females and normal individuals who do not receive PQ but that the distribution in the decline of Hb shifts to the left, i.e. PQ induces greater absolute and fractional falls in Hb. The Hb cost of PQ depends on the dose with a doubling (from ~10% to ~20%) of the upper 95% CI fractional fall seen in Tanzanian patients [13], to an approximately sixfold higher difference in the upper 95% CI of the absolute fall in Hb (3.8 vs. 0.6 g/dL) in the MDA study in which many children received toxic doses of PQ [40]. The latter study is a salutary reminder of the toxicity of high mg/kg PQ doses, possibly augmented by sulphadoxine, when given to G6PDd A- children [40]. One key group lacking PQ data is young, moderately anaemic (Hb < 8 g/dL) children with acute uncomplicated falciparum malaria.

### Background anaemia in African children

In African children, anaemia is common and its aetiology is frequently multifactorial [67]. The rates of background anaemia in Africa are high (e.g. 40–60%) across all malaria transmission intensities [68–73], but tend to be lower in low-transmission settings [69, 72]. Anaemia is inversely related to age in healthy individuals, asymptomatic falciparum carriers and malaria patients independent of malaria transmission intensity [68, 74, 75]. Table 3 shows Hb data in children aged <5 years from a community survey in the Kenyan highlands (low transmission) [68], and Table 4 shows Hb data in *P. falciparum*-infected patients of all ages from several African countries (database used in this analysis).

**Table 3:** Haemoglobin concentrations in g/dL in healthy children aged 6 months to 4 years from low-transmission areas in western Kenya [68], where only 0.3% (5/1697) were polymerase chain reaction (PCR) positive for *P. falciparum* in the community

Age (years)	1st	5th	10th	25th	Median	75th	90th	95th	99th	No.
<1	6.1	7.8	8.1	9.3	10.5	11.0	12.0	12.3	13.0	37
1	5.9	6.5	7.3	9.2	10.2	10.9	11.6	12.1	13.3	59
2	5.8	7.7	8.7	9.7	10.5	11.3	12.7	13.0	14.0	73
3	6.8	8.4	9.1	9.9	11.4	12.15	12.8	13.2	14.0	80
4	5.3	9	9.7	10.9	12.0	12.7	13.4	13.6	14.8	79

**Table 4:** Baseline haemoglobin concentrations in g/dL in patients of both sexes with acute uncomplicated falciparum malaria enrolled in trials in several African countries

Age (years)	1st	5th	10th	25th	Median	75th	90th	95th	99th	No.
0.5 to <1	4.8	5.5	6.0	6.9	8.2	9.7	10.7	11.7	12.8	1693
1	4.8	5.9	6.4	7.4	8.9	10.2	11.5	12.1	13.6	3247
2	4.8	5.9	6.7	7.8	9.4	10.7	11.7	12.7	14.0	2262
3	5.0	6.1	7.1	8.6	9.9	11.2	12.4	12.8	14.2	1838
4	5.0	6.3	7.3	8.9	10.2	11.5	12.5	13.2	14.3	1528
5	5.5	7.1	7.5	9.1	10.5	11.8	12.8	13.3	14.1	602
6	5.5	7.1	7.6	9.0	10.3	11.6	12.6	13	14.3	429
7	5.0	7.1	7.8	9.3	10.6	11.9	13.0	13.8	15.1	357
8	5.1	6.8	7.7	9.4	10.9	12.1	13.2	14.0	15.5	297
9	6.3	7.5	8.6	9.7	11.0	12.5	13.2	13.6	14.8	232
10	6.7	7.7	8.9	10.1	11.3	12.7	13.8	14.2	15.1	227
11	6.8	7.4	8.9	10	11.6	12.7	14.0	14.8	15.5	133
12	6.4	8.2	9.4	10.5	11.6	12.9	14.0	14.8	15.8	100
13	6.5	7.8	8.5	10.1	11.5	12.6	14.0	15.1	15.4	57
14	7.1	7.9	9.2	10.4	11.6	12.8	13.7	14.3	15.5	62
15	7.8	8.0	9.0	9.7	11.1	12.5	14.4	15.2	15.6	33
16	8.0	8.4	9.2	10.5	12.0	13.5	14.7	16.1	17.3	33
17	7.8	7.8	8.6	10.2	12.0	13.6	15.8	15.9	16.1	24
≥18	6.7	8.2	9.1	10.9	12.3	13.9	15.1	16	17.3	574

Younger children would be the prime recipients of SLDPQ in a programme that targets patients with symptomatic malaria. Although their median Hb concentrations at presentation may seem adequate (Table 4), a sizeable minority will have moderate anaemia and a small number will have severe anaemia (Hb < 5 g/dL). Younger children treated with ACT suffer greater post-treatment falls in Hb compared to older children. From our database, children aged <5 years had significantly lower Hb concentrations at baseline (9.3 vs. 10.9 g/dL) and D7 (8.6 vs. 10.5 g/dL) compared to children aged 5–11 years (*p* values < 0.001).

The risks of *P. falciparum*-infected children experiencing PQ-induced AHA and/or clinically significant anaemia (CSA), including profound anaemia (Hb < 4 g/dL) and severe anaemia (Hb < 5 g/dL) with features of severe malaria, are unknown. Limited data from a trial comparing ACTs [42] and a trial comparing SP vs. atovaquone-proguanil [76] suggest risk factors for a blood transfusion were: (i) high parasite biomass in ACT-treated children (clinical indication was decompensated anaemia), and, for the SP-treated children (clinical indications were Hb <3 g/dL or the development of respiratory distress or extreme lethargy), (ii) pre-treatment Hb < 5 g/dL, (iii) younger age, and (iv) high parasite biomass.

#### Summary of SLDPQ risk and background anaemia

There are two main reasons to be cautious in setting the upper dose limit of SLDPQ. Firstly, all the PQ studies that have recruited G6PDd patients have had high baseline Hb concentrations; therefore, we cannot extrapolate with confidence these findings to young symptomatic anaemic *P. falciparum*-infected children who have lower Hb concentrations (Table 4) and, secondly, the initial reduction in Hb level in PQ-treated asymptomatic *P. falciparum* carriers is similar to uncomplicated falciparum malaria patients, with some individuals experiencing Hb drops >2 g/dL [36, 37].

Predicting the fall in Hb in young PQ-treated malaria patients is challenging, but based on: (i) the published data cited above, (ii) unpublished summarised post-treatment Hb dynamics data for African patients aged <5 years (kindly provided by the WorldWide Antimalarial Resistance Network), (iii) the inverse relationship between baseline Hb and post-treatment Hb decline, and (iv) avoiding the tendency to underplay the toxicity of SLDPQ, we hypothesise that an average population of *P. falciparum*-infected African children aged <5 years may experience Hb declines of: (i) 1 g/dL (median), (ii) 1.5 g/dL (lower interquartile range), and (iii) 3 g/dL (lower 5%).

On the other hand, Shekalaghe et al*.* provide some reassurance that toxic doses of PQ were tolerated well in apparently healthy Tanzanian children aged 1–12 years with Hb concentrations ≥8 g/dL [40]. Moreover, in areas of low malaria transmission, the rates of background anaemia are less, so the risk of SLDPQ-induced AHA should be less compared to areas of high transmission and high background rates of anaemia.

We adopted a risk-stratified approach to setting the dose of SLDPQ. Given the uncertainty of the haemolytic potential of PQ in very young *P. falciparum*-infected children aged 6 − 11 months and the need to be cautious, we decided to under-dose them and arbitrarily set a dose of 1.25 mg PQ base, for a median PQ dose of 0.16 mg/kg (i.e. ~60% of the WHO recommendation). For children aged 1–5 years, we set an upper limit of 0.4 mg/kg of PQ base but increased this upper limit to 0.5 mg/kg for older children because they have less post-treatment declines in Hb and appear to tolerate SLDPQ well.

## Methods

### Assembling the African anthropometric database

We obtained permission to download anthropometric databases from the Demographic Health Survey website (https://dhsprogram.com/), which include a broad range of socioeconomic, geographical and other randomly collected data; however, the anthropometric data are confined to girls and boys aged <60 months and women aged 15 − 45 years. Other databases obtained from academic researchers, Médecins Sans Frontières, France, and Epicentre, Paris, contained data from malaria patients and field surveys of individuals from malaria-endemic areas.

The first author approached individuals to explain the purpose of this project and requested anonymised anthropometric data. Later, haematological data were also sought. All those who freely gave data would be co-authors and were expected to provide feedback on this paper.

Data obtained from KEMRI-WT (Kenya) followed a formal request to a data access committee. We considered ethical approval unnecessary for this project, which was confirmed by the Oxford Tropical Research Ethics Committee.

#### Modelling the weight-for-age data

The raw weight-for-age data were modelled into a growth curve (Additional file 1: Figure S1) by excluding outlying weight-for-age data, which were defined as values falling outside the 1st and 99th centiles (as was done for the Cambodian database). Modelled weight-for-age centiles were obtained using the three-parameter Box–Cox power exponential distribution [77] and the centile growth curves smoothed using the cubic spline smoothing technique [78]. The modelling was performed in Stata using the ‘xriml’ macro Stata module [77] with a cubic spline.

The ages of children aged 6 − 11 months were expressed as a decimal of 1 year. Ages ≥12 months were rounded down to whole integers (e.g. 2 years and 5 months was rounded down to 2 years) and ages ≥18 years were grouped together. The mg base/kg dose of PQ received for a given age group was calculated as PQ dose/all the weights for that age group.

#### Data analysis and determining optimal age-dosing categories

Within each age-dosing group, we calculated the proportions of males and females who would receive therapeutic doses for a given age in years as well as for the age-dosing group overall. Male–female differences were compared by Fisher’s exact test, differences in the distribution of skewed data were compared using Mann Whitney U test and mean differences in normally distributed data were compared using an unpaired *t*-test. A *p* value ≤0.05 was considered significant.

PQ doses of 1–15 mg were tested across different age groups and the results analysed. The final age categories were selected based on: (i) the need to be especially cautious in children aged 6 − 11 months, (ii) the mg base/kg dose received, (iii) the proportions receiving a therapeutic dose, (iv) how well a given dosing band fitted in with the next dosing band, and (v) a desire to minimise the number of dosing groups and to use the same tablet strengths as those in the Cambodian regimen (i.e. 2.5, 5, 7.5 and 15 mg of PQ base).

## Results

### Features of the anthropometric database

**Fig. 1:**
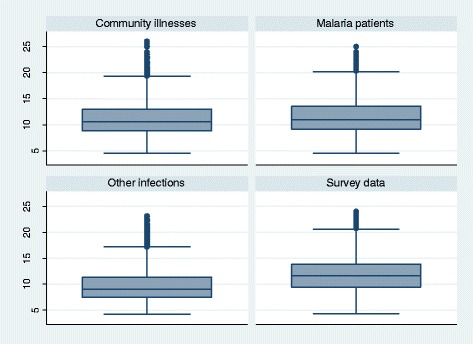
Box plots of modelled weights in children aged less than five years by health status

The modelled database numbered 661,979 individuals aged ≥6 months. The female/male ratio is 2:1 [missing data = 95 (0.01%)] and the rural/urban ratio is 2.6:1. Individuals aged 6–11 months, 1 − 4 years, 5–14 years and ≥15 years represent approximately 6%, 38%, 6% and 50% of the database. The Demographic Health Survey data accounted for 83.0% (*n* = 549,127) of the database whilst malaria (*n* = 28,466), other infections (*n* = 30,009) and miscellaneous data from rural clinics (*n* = 54,377) accounted for 4.3%, 4.5%, and 8.2%, respectively. The distributions of these categories by countries are shown in Additional file 2: Table S1. The weight distributions in children aged ≤5 years with and without malaria overlapped substantially (Fig. 1).

### Dosing regimen

There are five age-dosing groups (Table 5). Overall, the model predicts that ~94–99% of patients in dosing bands from 1 year upwards would receive a PQ dose within the defined therapeutic range, and that their median doses would range from 0.23 to 0.29 mg/kg of PQ base. The median dose for children aged 6–11 months is 0.16 mg/kg. Based on our minimally efficacious transmission-blocking dose of 0.15 mg/kg PQ, 27% of infants will be under-dosed but this falls to 3.3% (1,322/39,886) if using the minimum dose of 0.125 mg/kg of PQ base, as reported by Dicko et al*.*

**Table 5:** The proposed age-based dosing regimen showing the proportions of individuals who would receive a subtherapeutic, therapeutic or supratherapeutic dose and the corresponding mg base/kg doses of primaquine (PQ)

	PQ dose (mg base)	Subtherapeutic dose <0.15 mg/kg, *n* (%)	Therapeutic dose 0.15–0.5 mg/kg, *n* (%)	Supratherapeutic dose >0.5 mg/kg, *n* (%)	Modelled weights by centile (kg)			PQ dose by centile (mg base/kg)		
					1st	Median	99th	1st	Median	99th
0.5 to <1 (*n* = 39,886)	1.25	10,778 (27.0)	29,180 (73.2)	0	5.0	7.6	10.8	0.12	0.16	0.25
1–5 (*n* = 261,036)	2.5	15,744 (6.0)	244,537 (93.7)	755 (0.3)	6.8	12.0	19.2	0.13	0.21	0.37
6–9 (*n* = 20,770)	5	80 (0.4)	20,690 (99.6)	0	13.2	20.0	31.2	0.16	0.25	0.38
10–14 (*n* = 12,155)	7.5	69 (0.6)	12,086 (99.4)	0	20.0	29.0	49.0	0.15	0.26	0.38
≥15 (*n* = 328,132)	15	469 (0.14)	327,620 (99.8)	43 (0.01)	37.8	55.3	90.2	0.17	0.27	0.40

*PQ* primaquine

### Primaquine dose breakdown by individual ages and sex

**Fig. 2:**
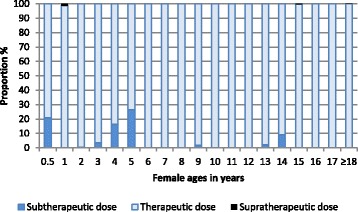
Histogram showing the proportions of females who would receive a subtherapeutic, therapeutic or supratherapeutic dose of primaquine

**Fig. 3:**
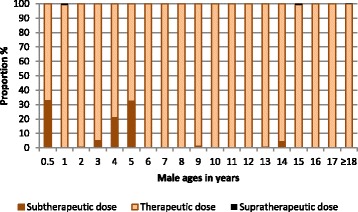
Histogram showing the proportions of males who would receive a subtherapeutic, therapeutic or supratherapeutic dose of primaquine

The proportions of females and males predicted to receive subtherapeutic, supratherapeutic and therapeutic doses are shown in Figs. 2 and 3, respectively. Rates of supratherapeutic dosing are low, a maximum of 1.3% in females aged 1 year. Age groups at risk of under-dosing exceeding 5% are females and males aged 6 to <12 months, 4 and 5 years, and females aged 14 years. Altogether, 73.5% to 100% of females (Fig. 2) and 67.5% to 100% of males (Fig. 3) would receive a therapeutic dose.

**Fig. 4:**
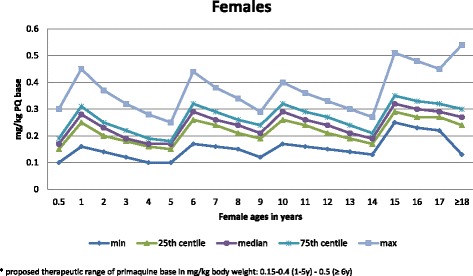
The mg base/kg dose of primaquine (PQ) by centile that females would receive as a function of age. The proposed therapeutic range of PQ base in mg/kg body weight is 0.15–0.4 (1–5 years) to 0.5 (≥6 years). PQ primaquine

**Fig. 5:**
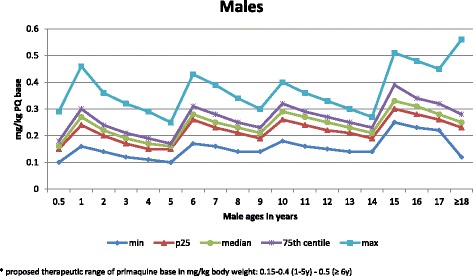
The mg base/kg dose of primaquine (PQ) base by centile that males would receive as a function of age. The proposed therapeutic range of PQ base in mg/kg body weight is 0.15–0.4 (1–5 years) to 0.5 (≥6 years). PQ primaquine

The median mg base/kg dose of PQ base ranged from 0.17 to 0.32 in females (Fig. 4) and from 0.17 to 0.33 in males (Fig. 5). The age groups with the lowest median mg/kg dose were 6–11 months, 4 years, and 5 years (0.17), and 3 years and 14 years (0.19) for females, and 6–11 months and 5 years (0.16), 4 years (0.17), and 3 years (0.19) for males.

## Discussion

This age-dosing SLDPQ regimen for SSA was developed from an anthropometric database of more than 660,000 individuals from across the continent. It has five dosing groups and how optimised they are will be tested in a large randomised trial to assess its safety in G6PDd and G6PD normal African children with acute uncomplicated falciparum malaria (ISRCTN11594437). If this trial shows acceptable safety and adequate PK profiles, it will provide substantial reassurance to African ministries of health in accelerating the uptake of SLDPQ.

These data overlapped substantially with data from malaria patients and other survey data conducted in predominantly rural areas. Thus, our database is representative of target populations for SLDPQ. Although the WHO currently recommends that SLDPQ be used in symptomatic malaria patients, it is likely to be used as an elimination tool in asymptomatic parasite carriers, who contribute substantially to malaria transmission [79].

Setting the upper therapeutic range of PQ is crucial for safety and challenging in the absence of a robust dose–response relationship of PQ PK and Hb decline. In contrast to our approach for Cambodia, where malaria is mostly a disease of adults and where background rates of anaemia and malaria-related anaemia are lower [18], we placed much emphasis on the risk of young paediatric patients developing CSA following ACT + PQ because they have lower pre-treatment Hb concentrations and greater falls in Hb, and so a higher risk of CSA compared to older children. In addition, two studies support high parasite biomass as another risk factor for CSA [42, 76].

Therefore, we decided to be cautious and deliberately under-dose infants with 1.25 mg of PQ base, resulting in a mg/kg dose range of 0.1–0.3 (median of 0.16). In this scenario, ~60% of infants would receive less than the WHO-recommended PQ dose of 0.25 mg/kg. What effect this would have on mosquito infectivity is unknown and more research is needed to establish the dose–response (i.e. PQ PK–infectivity) relationship and refine the minimum PQ dose.

We set a minimum PQ dose of 0.15 mg/kg, based on data from the field experience of MDA in Cambodia [80], which is higher than the 0.125 mg/kg suggested by Dicko et al. in their mosquito infectivity experiments [81]. Which of the two is the more accurate value is unknown. Our threshold predicts ~25% under-dose rate in infants compared to 3% if we use the threshold of Dicko et al. This emphasises the need to define accurately the minimum transmission-blocking dose and to consider how much of a transmission reservoir such young children represent and, therefore, the need to give them SLDPQ.

Much emphasis and anxiety have been placed on the potential dangers of PQ-induced AHA in treated *P. falciparum*-infected G6PDd individuals but there are many other factors that may play an important role in post-treatment Hb dynamics and the risk of post-treatment CSA, such as sickle-cell trait/disease, alpha-thalassaemia, HIV infection, hookworm infection, vitamin A deficiency, poor nutritional state, schistosomiasis, baseline parasitaemia and Hb, and PQ PK [42, 67, 73, 76, 82].

Insights will be forthcoming from our safety trial of SLDPQ and from the large multicentre TRACT study (Transfusion and treatment of severe anaemia in African children trial [83]), which is designed to address optimal blood transfusion strategies in children admitted to hospital with Hb ≤6 g/dL. Data from both trials will inform strategies aimed at identifying children at risk of CSA before they are treated with ACT + SLDPQ and how this may be best achieved in challenging settings, e.g. small remote clinics that may not have accurate point-of-care tests to measure Hb concentration or the time or means to quantify parasitaemias.

Another key safety issue is the haemolytic effect of SLDPQ in individuals carrying the severe G6PDd Santamaria and Mediterranean variants. Limited data in *P. falciparum*-infected Cambodians with G6PDd Viangchan (median G6PD activity 0.8 U/g Hb [17, 46]) are encouraging, but are only partially applicable to Africa. Fractional declines in Hb on D7 overlapped in G6PDd and G6PD normal patients (mostly adults) who were treated with DHAPP + SLDPQ or DHAPP alone (L. Desoley, unpublished data). One reassuring aspect of our age-based regimen is that we have restricted the upper limits of PQ and the proportions of individuals receiving supratherapeutic doses is very small, <1.4% in those aged 1 or 15 years.

Our SLDPQ regimen has five dosing bands and the first (6–11 months) has the most restricted age limits. Anecdotally, concern is expressed that age-based dosing is intrinsically inaccurate because patients or guardians are unable to report ages accurately and birth certificates or ‘Road to Health’ cards may not be widely available in some areas. This was not an issue in the MDA of ASAQ during the Ebola outbreak in which 98% (983/1005) of individuals or guardians in Monrovia were able to tell their ages and take the correct dose of ASAQ [20]. Nevertheless, this is an aspect that needs to be considered when SLDPQ is deployed.

This SLDPQ regimen shares the same tablet strengths (aside from the 6–11 months age band) as the one designed for Cambodia [16]. This overlap in regimens between Africa and Cambodia (and by extension the Greater Mekong Subregion) may reassure the pharmaceutical industry regarding the market for SLDPQ. Indeed, the 2012 WHO recommendation was global. Moreover, if the same table strengths could be used in regimens for *P. vivax* radical cure, this should stimulate the pharmaceutical industry to produce 2.5- and 5-mg tablet strengths and paediatric formulations. This would have a positive effect on access. Substantial evidence on the safety of SLDPQ should be forthcoming in the next 2 years from our trial and other studies, and should allay anxieties that pharmaceutical companies may have regarding PQ toxicity and their liability.

Our analysis has limitations. Similar to others [19, 84], our database was dominated by children aged <5 years and women of childbearing age because most data from the Demographic Health Survey and malaria patients, especially in SSA, are from these groups. Although children aged 5–17 years represented 12% of the total database, each individual age group contained >2500 individuals (except ages 13 and 14 years).

The dosing regimen is not generalisable to countries outside of SSA and, like the Cambodian regimen, does not dovetail nicely with the age–weight dosing categories of commonly used ACTs. DHAPP has eight weight-based dosing bands with an emphasis on children who weigh <25 kg to ensure they receive sufficient dihydroartemisinin. AL, currently the most widely used ACT in Africa, has four weight-based dosing bands and ASAQ has four age-based bands (2–11 months, 1–5 years, 6–13 years and ≥14 years) that share two of the five age-based bands of our SLDPQ regimen. Suitable training would be essential before SLDPQ is implemented by programmes.

We designed this SLDPQ regimen based on current but incomplete knowledge, especially PQ PK in young children. Goncalves et al. have contributed useful data in asymptomatic falciparum carriers [26] and we hope to close the PK gap further. With this new knowledge and, possibly, the eventual definition of the dose–response relationship between PQ’s oxidative metabolites and haemolysis and mosquito infectivity, our proposed SLDPQ regimen may need to be amended.

## Conclusions

This age-based regimen of SLDPQ was designed to block *P. falciparum* transmission in SSA and will be tested for safety in a large trial in Uganda and the Democratic Republic of Congo in 2017. Depending on the results, this dose regimen may require fine-tuning but could also be readily adopted by national malaria control programmes.

## Additional files

Additional file 1: Weight-for-age growth curves. (PDF 1202 kb)

Additional file 2: Table S1. Data type by country. (DOCX 14 kb)

## Abbreviations

ACT: Artemisinin-based combination therapy
AHA: Acute haemolytic anaemia
AL: Artemether-lumefantrine
AQ: Amodiaquine
AS: Artesunate
ASAQ: Artesunate-amodiaquine
CI: Confidence interval
CSA: Clinically significant anaemia
D: Day
DHAPP: Dihydroartemisinin-piperaquine
G6PDd: Glucose-6-phosphate dehydrogenase deficiency
Hb: Haemoglobin
MDA: Mass drug administration
metHb: Methaemoglobin
MQ: Mefloquine
PD: Pharmacodynamics
PK: Pharmacokinetics
PKPD: Pharmacokinetics–pharmacodynamics
PQ: Primaquine
SLDPQ: Single low-dose primaquine
SP: Sulphadoxine-pyrimethamine
SSA: Sub-Saharan Africa
WHO: World Health Organization

## References

[1] Noedl H, Se Y, Schaecher K, Smith BL, Socheat D, Fukuda MM, Artemisinin Resistance in Cambodia 1 Study C (2008). Evidence of artemisinin-resistant malaria in western Cambodia. N Engl J Med.

[2] Dondorp AM, Nosten F, Yi P, Das D, Phyo AP, Tarning J (2009). Artemisinin resistance in *Plasmodium falciparum* malaria. N Engl J Med.

[3] Ariey F, Witkowski B, Amaratunga C, Beghain J, Langlois AC, Khim N (2014). A molecular marker of artemisinin-resistant *Plasmodium falciparum* malaria. Nature.

[4] Phyo AP, Nkhoma S, Stepniewska K, Ashley EA, Nair S, McGready R (2012). Emergence of artemisinin-resistant malaria on the western border of Thailand: a longitudinal study. Lancet.

[5] Hien TT, Thuy-Nhien NT, Phu NH, Boni MF, Thanh NV, Nha-Ca NT (2012). In vivo susceptibility of *Plasmodium falciparum* to artesunate in Binh Phuoc Province. Vietnam Malar J.

[6] Ashley EA, Dhorda M, Fairhurst RM, Amaratunga C, Lim P, Suon S (2014). Spread of artemisinin resistance in *Plasmodium falciparum* malaria. N Engl J Med.

[7] Tun KM, Imwong M, Lwin KM, Win AA, Hlaing TM, Hlaing T (2015). Spread of artemisinin-resistant *Plasmodium falciparum* in Myanmar: a cross-sectional survey of the K13 molecular marker. Lancet Infect Dis.

[8] Menard D, Khim N, Beghain J, Adegnika AA, Shafiul-Alam M, Amodu O (2016). A worldwide map of *Plasmodium falciparum* K13-propeller polymorphisms. N Engl J Med.

[9] White NJ, Qiao LG, Qi G, Luzzatto L (2012). Rationale for recommending a lower dose of primaquine as a *Plasmodium falciparum* gametocytocide in populations where G6PD deficiency is common. Malar J..

[10] Binka F, Graves PM, Marsh K, Leke RF (2012). Malaria Policy Advisory Committee to the WHO: conclusions and recommendations of September 2012 meeting. Malar J..

[11] Chen I, Poirot E, Newman M, Kandula D, Shah R, Hwang J (2015). An assessment of the supply, programmatic use, and regulatory issues of single low-dose primaquine as a *Plasmodium falciparum* gametocytocide for sub-Saharan Africa. Malar J..

[12] Bancone G, Chowwiwat N, Somsakchaicharoen R, Poodpanya L, Moo PK, Gornsawun G, 3 (2016). Single low dose primaquine (0.25 mg/kg) does not cause clinically significant haemolysis in G6PD deficient subjects. PLoS One.

[13] Mwaiswelo R, Ngasala B, Jovel I, Aydin-Schmidt B, Gosling R, Premji Z, 1 (2016). Adding a single low-dose of primaquine (0.25 mg/kg) to artemether-lumefantrine did not compromise treatment outcome of uncomplicated *Plasmodium falciparum* malaria in Tanzania: a randomized, single-blinded clinical trial. Malar J.

[14] Poirot E, Soble A, Ntshalintshali N, Mwandemele A, Mkhonta N, Malambe C (2016). Development of a pharmacovigilance safety monitoring tool for the rollout of single low-dose primaquine and artemether-lumefantrine to treat *Plasmodium falciparum* infections in Swaziland: a pilot study. Malar J.

[15] The World Health Organization. Guidelines for the Treatment of Malaria. 3rd edition. 2015. p 42.

[16] Leang R, Khu NH, Mukaka M, Debackere M, Tripura R, Kheang ST (2016). An optimised age-based dosing regimen for single low-dose primaquine for blocking malaria transmission in Cambodia. BMC Med.

[17] Kim S, Nguon C, Guillard B, Duong S, Chy S, Sum S (2011). Performance of the CareStart G6PD deficiency screening test, a point-of-care diagnostic for primaquine therapy screening. PLoS One.

[18] Khim N, Benedet C, Kim S, Kheng S, Siv S, Leang R (2013). G6PD deficiency in *Plasmodium falciparum* and *Plasmodium vivax* malaria-infected Cambodian patients. Malar J..

[19] Taylor W, Terlouw DJ, Olliaro PL, White NJ, Brasseur P, ter Kuile FO (2006). Use of weight-for-age-data to optimize tablet strength and dosing regimens for a new fixed-dose artesunate-amodiaquine combination for treating falciparum malaria. Bull World Health Organ.

[20] Kuehne A, Tiffany A, Lasry E, Janssens M, Besse C, Okonta C (2016). Impact and lessons learned from mass drug administrations of malaria chemoprevention during the Ebola outbreak in Monrovia, Liberia, 2014. PLoS One.

[21] Ameh S, Welaga P, Kabiru CW, Ndifon W, Ikpeme B, Nsan E (2015). Factors associated with appropriate home management of uncomplicated malaria in children in Kassena-Nankana district of Ghana and implications for community case management of childhood illness: a cross-sectional study. BMC Public Health..

[22] O'Connell KA, Samandari G, Phok S, Phou M, Dysoley L, Yeung S (2012). ‘Souls of the ancestor that knock us out’ and other tales. A qualitative study to identify demand-side factors influencing malaria case management in Cambodia. Malar J..

[23] Yeung S, Van Damme W, Socheat D, White NJ, Mills A (2008). Access to artemisinin combination therapy for malaria in remote areas of Cambodia. Malar J..

[24] Xu JW, Xu QZ, Liu H, Zeng YR (2012). Malaria treatment-seeking behaviour and related factors of Wa ethnic minority in Myanmar: a cross-sectional study. Malar J..

[25] Marsh VM, Mutemi WM, Willetts A, Bayah K, Were S, Ross A (2004). Improving malaria home treatment by training drug retailers in rural Kenya. Trop Med Int Health.

[26] Goncalves BP, Pett H, Tiono AB, Murry D, Sirima SB, Niemi M, et al. Age, weight, and CYP2D6 genotype are major determinants of primaquine pharmacokinetics in African children. Antimicrob Agents Chemother. 2017;61(5). 10.1128/AAC.02590-16 PMC5404566 28289025

[27] Dick GW, Bowles RV (1947). The value of plasmoquine as a gametocide in sub-tertian malaria. Trans R Soc Trop Med Hyg.

[28] Mackerras MJ, Ercole QN (1949). Some observations on the action of quinine, atebrin, and plasmoquine on *Plasmodium vivax*. Trans R Soc Trop Med Hyg.

[29] Burgess RW, Bray RS (1961). The effect of a single dose of primaquine on the gametocytes, gametogony and sporogony of *Laverania falciparum*. Bull World Health Organ..

[30] Rieckmann KH, McNamara JV, Kass L, Powell RD (1969). Gametocytocidal and sporontocidal effects of primaquine upon two strains of *Plasmodium falciparum*. Mil Med.

[31] Dicko A, Brown JM, Diawara H, Baber I, Mahamar A, Soumare HM, et al. Primaquine to reduce transmission of *Plasmodium falciparum* malaria in Mali: a single-blind, dose-ranging, adaptive randomised phase 2 trial. Lancet Infect Dis. 2016;16:674-84. 10.1016/S1473-3099(15)00479-X PMC10583596 26906747

[32] White NJ, Ashley EA, Recht J, Delves MJ, Ruecker A, Smithuis FM (2014). Assessment of therapeutic responses to gametocytocidal drugs in *Plasmodium falciparum* malaria. Malar J..

[33] Churcher TS, Bousema T, Walker M, Drakeley C, Schneider P, Ouedraogo AL (2013). Predicting mosquito infection from *Plasmodium falciparum* gametocyte density and estimating the reservoir of infection. Elife..

[34] Jeffery GM, Eyles DE (1955). Infectivity to mosquitoes of *Plasmodium falciparum* as related to gametocyte density and duration of infection. Am J Trop Med Hyg.

[35] Pethleart A, Prajakwong S, Suwonkerd W, Corthong B, Webber R, Curtis C (2004). Infectious reservoir of *Plasmodium* infection in Mae Hong Son Province, north-west Thailand. Malar J..

[36] Goncalves BP, Tiono AB, Ouedraogo A, Guelbeogo WM, Bradley J, Nebie I (2016). Single low dose primaquine to reduce gametocyte carriage and *Plasmodium falciparum* transmission after artemether-lumefantrine in children with asymptomatic infection: a randomised, double-blind, placebo-controlled trial. BMC Med..

[37] Okebe J, Bousema T, Affara M, Di Tanna GL, Dabira E, Gaye A, et al. The gametocytocidal efficacy of different single doses of primaquine with dihydroartemisinin-piperaquine in asymptomatic parasite carriers in the Gambia: a randomized controlled trial. EBioMedicine. 2016;13:348–55. 10.1016/j.ebiom.2016.10.032 PMC5264436 27825738

[38] Ashley EA, Recht J, White NJ (2014). Primaquine: the risks and the benefits. Malar J..

[39] Eziefula AC, Bousema T, Yeung S, Kamya M, Owaraganise A, Gabagaya G, et al. Single dose primaquine for clearance of *Plasmodium falciparum* gametocytes in children with uncomplicated malaria in Uganda: a randomised, controlled, double-blind, dose-ranging trial. Lancet Infect Dis. 2013;14(2):130–9 10.1016/S1473-3099(13)70268-8 24239324

[40] Shekalaghe SA, ter Braak R, Daou M, Kavishe R, van den Bijllaardt W, van den Bosch S (2010). In Tanzania, hemolysis after a single dose of primaquine coadministered with an artemisinin is not restricted to glucose-6-phosphate dehydrogenase-deficient (G6PD A-) individuals. Antimicrob Agents Chemother.

[41] Creek D, Bigira V, Arinaitwe E, Wanzira H, Kakuru A, Tappero J (2010). Increased risk of early vomiting among infants and young children treated with dihydroartemisinin-piperaquine compared with artemether-lumefantrine for uncomplicated malaria. Am J Trop Med Hyg.

[42] Onyamboko AM, Fanello CI, Wongsaen K, Tarning J, Cheah PY, Tshefu KA, et al. A randomized comparison of the efficacy and tolerability of three artemisinin-based combination treatments for children with acute falciparum malaria in The Democratic Republic of Congo. Antimicrob Agents Chemother. 2014;58(9):5528–36 10.1128/AAC.02682-14 PMC4135835 25001306

[43] Falade C, Manyando C (2009). Safety profile of Coartem: the evidence base. Malar J.

[44] Schramm B, Valeh P, Baudin E, Mazinda CS, Smith R, Pinoges L (2013). Tolerability and safety of artesunate-amodiaquine and artemether-lumefantrine fixed dose combinations for the treatment of uncomplicated *Plasmodium falciparum* malaria: two open-label, randomized trials in Nimba County. Liberia Malar J..

[45] Anstey NM, Hassanali MY, Mlalasi J, Manyenga D, Mwaikambo ED (1996). Elevated levels of methaemoglobin in Tanzanian children with severe and uncomplicated malaria. Trans R Soc Trop Med Hyg.

[46] Kheng S, Muth S, Taylor WR, Tops N, Kosal K, Sothea K (2015). Tolerability and safety of weekly primaquine against relapse of *Plasmodium vivax* in Cambodians with glucose-6-phosphate dehydrogenase deficiency. BMC Med..

[47] Luzzatto L (2010). The rise and fall of the antimalarial Lapdap: a lesson in pharmacogenetics. Lancet.

[48] Peto TJ, Kloprogge SE, Tripura R, Nguon C, Sanann N, Yok S (2016). History of malaria treatment as a predictor of subsequent subclinical parasitaemia: a cross-sectional survey and malaria case records from three villages in Pailin, western Cambodia. Malar J..

[49] Luzzatto L, Seneca E (2014). G6PD deficiency: a classic example of pharmacogenetics with on-going clinical implications. Br J Haematol.

[50] Beutler E (1996). G6PD: population genetics and clinical manifestations. Blood Rev.

[51] Shah SS, Macharia A, Makale J, Uyoga S, Kivinen K, Craik R (2014). Genetic determinants of glucose-6-phosphate dehydrogenase activity in Kenya. BMC Med Genet..

[52] Howes RE, Dewi M, Piel FB, Monteiro WM, Battle KE, Messina JP (2013). Spatial distribution of G6PD deficiency variants across malaria-endemic regions. Malar J..

[53] Okebe J, Amambua-Ngwa A, Parr J, Nishimura S, Daswani M, Takem EN (2014). The prevalence of glucose-6-phosphate dehydrogenase deficiency in Gambian school children. Malar J..

[54] Clark TG, Fry AE, Auburn S, Campino S, Diakite M, Green A (2009). Allelic heterogeneity of G6PD deficiency in West Africa and severe malaria susceptibility. Eur J Hum Genet.

[55] Tishkoff SA, Varkonyi R, Cahinhinan N, Abbes S, Argyropoulos G, Destro-Bisol G (2001). Haplotype diversity and linkage disequilibrium at human G6PD: recent origin of alleles that confer malarial resistance. Science.

[56] May J, Meyer CG, Grossterlinden L, Ademowo OG, Mockenhaupt FP, Olumese PE (2000). Red cell glucose-6-phosphate dehydrogenase status and pyruvate kinase activity in a Nigerian population. Trop Med Int Health.

[57] LaRue N, Kahn M, Murray M, Leader BT, Bansil P, McGray S (2014). Comparison of quantitative and qualitative tests for glucose-6-phosphate dehydrogenase deficiency. Am J Trop Med Hyg.

[58] Adu-Gyasi D, Asante KP, Newton S, Dosoo D, Amoako S, Adjei G (2015). Evaluation of the diagnostic accuracy of CareStart G6PD deficiency Rapid Diagnostic Test (RDT) in a malaria endemic area in Ghana. Africa. PloS One..

[59] Ouattara AK, Yameogo P, Diarra B, Obiri-Yeboah D, Yonli A, Compaore TR (2016). Molecular heterogeneity of glucose-6-phosphate dehydrogenase deficiency in Burkina Faso: G-6-PD betica selma and santamaria in people with symptomatic malaria in Ouagadougou. Mediterr J Hematol Infect Dis.

[60] Beutler E, Kuhl W, Saenz GF, Rodriguez W (1991). Mutation analysis of glucose-6-phosphate dehydrogenase (G6PD) variants in Costa Rica. Hum Genet.

[61] Cittadella R, Civitelli D, Manna I, Azzia N, Di Cataldo A, Schiliro G (1997). Genetic heterogeneity of glucose-6-phosphate dehydrogenase deficiency in south-east Sicily. Ann Hum Genet.

[62] Shah SS, Rockett KA, Jallow M, Sisay-Joof F, Bojang KA, Pinder M (2016). Heterogeneous alleles comprising G6PD deficiency trait in West Africa exert contrasting effects on two major clinical presentations of severe malaria. Malar J..

[63] Saha N, Samuel AP, Omer A, Hoffbrand AV (1983). The inter- and intra-tribal distribution of red cell G6PD phenotypes in Sudan. Hum Hered.

[64] Saha N, Samuel AP, Omer A, Ahmed MA, Hussein AA, Gaddoura EN (1978). A study of some genetic characteristics of the population of the Sudan. Ann Hum Biol.

[65] Eziefula AC, Pett H, Grignard L, Opus S, Kiggundu M, Kamya MR (2014). Glucose-6-phosphate dehydrogenase status and risk of hemolysis in *Plasmodium falciparum*-infected African children receiving single-dose primaquine. Antimicrob Agents Chemother.

[66] Shekalaghe S, Drakeley C, Gosling R, Ndaro A, van Meegeren M, Enevold A (2007). Primaquine clears submicroscopic *Plasmodium falciparum* gametocytes that persist after treatment with sulphadoxine-pyrimethamine and artesunate. PLoS One.

[67] Calis JC, Phiri KS, Faragher EB, Brabin BJ, Bates I, Cuevas LE (2008). Severe anemia in Malawian children. N Engl J Med.

[68] Noland GS, Ayodo G, Abuya J, Hodges JS, Rolfes MA, John CC (2012). Decreased prevalence of anemia in highland areas of low malaria transmission after a 1-year interruption of transmission. Clin Infect Dis.

[69] Carter JY, Lema OE, Mukunza HK, Varia HN, Munyere AS, Watkins WM (1999). Prevalence of anaemia in patients attending an outpatient clinic in western Rift Valley in Kenya during a low malaria season. East Afr Med J.

[70] Sangweme DT, Midzi N, Zinyowera-Mutapuri S, Mduluza T, Diener-West M, Kumar N (2010). Impact of schistosome infection on *Plasmodium falciparum* malariometric indices and immune correlates in school age children in Burma Valley, Zimbabwe. PLoS Negl Trop Dis..

[71] Zucker JR, Perkins BA, Jafari H, Otieno J, Obonyo C, Campbell CC (1997). Clinical signs for the recognition of children with moderate or severe anaemia in western Kenya. Bull World Health Organ..

[72] Akhwale WS, Lum JK, Kaneko A, Eto H, Obonyo C, Bjorkman A (2004). Anemia and malaria at different altitudes in the western highlands of Kenya. Acta Trop.

[73] Tsang BL, Sullivan KM, Ruth LJ, Williams TN, Suchdev PS (2014). Nutritional status of young children with inherited blood disorders in western Kenya. Am J Trop Med Hyg.

[74] Ardiet DL, Graz B, Szeless T, Mauris A, Falquet J, Doumbo OK (2014). Patterns of malaria indices across three consecutive seasons in children in a highly endemic area of West Africa: a three times-repeated cross-sectional study. Malar J..

[75] Diakite M, Miura K, Diouf A, Konate D, Keita AS, Doumbia S (2016). Hematological indices in Malian children change significantly during a malaria season and with increasing age: implications for malaria epidemiological studies. Am J Trop Med Hyg.

[76] Mulenga M, Malunga F, Bennett S, Thuma PE, Shulman C, Fielding K, et al. A randomised, double-blind, placebo-controlled trial of atovaquoneproguanil vs. sulphadoxine-pyrimethamine in the treatment of malarial anaemia in Zambian children. Trop Med Int Health. 2006;11(11):1643–52 10.1111/j.1365-3156.2006.01726.x 17054743

[77] Wright EM, Royston P (1997). Simplified estimation of age-specific reference intervals for skewed data. Stat Med.

[78] Rigby RA, Stasinopoulos DM (2004). Smooth centile curves for skew and kurtotic data modelled using the Box–Cox power exponential distribution. Stat Med.

[79] Okell LC, Bousema T, Griffin JT, Ouedraogo AL, Ghani AC, Drakeley CJ (2012). Factors determining the occurrence of submicroscopic malaria infections and their relevance for control. Nat Commun..

[80] Song J, Socheat D, Tan B, Dara P, Deng C, Sokunthea S (2010). Rapid and effective malaria control in Cambodia through mass administration of artemisinin-piperaquine. Malar J..

[81] Dicko A, Brown JM, Diawara H, Baber I, Mahamar A, Soumare HM (2016). Primaquine to reduce transmission of *Plasmodium falciparum* malaria in Mali: a single-blind, dose-ranging, adaptive randomised phase 2 trial. Lancet Infect Dis.

[82] Midzi N, Sangweme D, Zinyowera S, Mapingure MP, Brouwer KC, Munatsi A (2008). The burden of polyparasitism among primary schoolchildren in rural and farming areas in Zimbabwe. Trans R Soc Trop Med Hyg.

[83] Mpoya A, Kiguli S, Olupot-Olupot P, Opoka RO, Engoru C, Mallewa M (2015). Transfusion and treatment of severe anaemia in African children (TRACT): a study protocol for a randomised controlled trial. Trials..

[84] Hayes DJ, van Buuren S, Ter Kuile FO, Stasinopoulos DM, Rigby RA, Terlouw DJ (2015). Developing regional weight-for-age growth references for malaria-endemic countries to optimize age-based dosing of antimalarials. Bull World Health Organ.

