# Supplementary figures and images for: Single low-dose primaquine for blocking transmission of Plasmodium falciparum malaria – a proposed model-derived age-based regimen for sub-Saharan Africa
Source: BMC Med. 2018 Jan 18;16:11. doi: 10.1186/s12916-017-0990-6 (PMC5774032; doi:10.1186/s12916-017-0990-6)

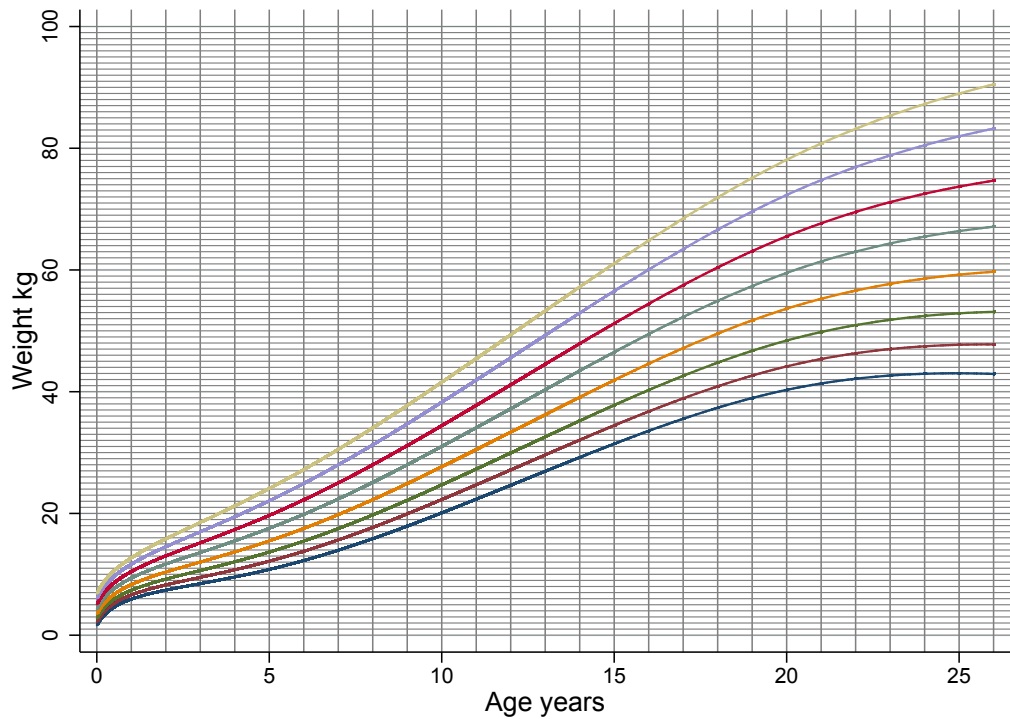

Supplement: Supplementary file 1 — Weight-for-age growth curves. (PDF 1202 kb) [file 12916_2017_990_MOESM1_ESM.pdf]
