# Supplementary material for: Single low-dose primaquine for blocking transmission of Plasmodium falciparum malaria – a proposed model-derived age-based regimen for sub-Saharan Africa
Source: BMC Med. 2018 Jan 18;16:11. doi: 10.1186/s12916-017-0990-6 (PMC5774032; doi:10.1186/s12916-017-0990-6)
Supplement: Supplementary file 2 — Data type by country. (DOCX 14 kb) [file 12916_2017_990_MOESM2_ESM.docx]

**Table S1** Data type by country

| **Country** | **Healthy individuals** | **Malaria** | **Other infections** | **Miscellaneous** | **Total** |
| --- | --- | --- | --- | --- | --- |
| Benin | 27,379 | 0 | 0 | 0 | 27,379 |
| Burkina Faso | 14,722 | 1,148 | 0 | 0 | 15,870 |
| Burundi | 7,888 | 0 | 0 | 0 | 7,888 |
| Cameroon | 13,240 | 140 | 0 | 0 | 13,380 |
| Chad | 21,388 | 0 | 0 | 0 | 21,388 |
| Congo | 73 | 296 | 0 | 0 | 369 |
| DRC | 19,061 | 975 | 22 | 0 | 20,058 |
| Ethiopia | 40,213 | 0 | 0 | 0 | 40,213 |
| Gabon | 9,227 | 243 | 0 | 0 | 9,470 |
| Ghana | 11,792 | 0 | 0 | 0 | 11,792 |
| Guinea | 7,890 | 0 | 0 | 0 | 7,890 |
| Ivory coast | 16,980 | 0 | 0 | 0 | 16,980 |
| Kenya | 47,807 | 8,834 | 28,096 | 54,377 | 139,114 |
| Liberia | 20,680 | 959 | 0 | 0 | 21,639 |
| Madagascar | 8,831 | 0 | 1,891 | 0 | 10,722 |
| Malawi | 12,534 | 438 | 0 | 0 | 12,972 |
| Mali | 9,850 | 786 | 0 | 0 | 10,636 |
| Mozambique | 23,277 | 270 | 0 | 0 | 23,547 |
| Nigeria | 62,815 | 0 | 0 | 0 | 62,815 |
| Rwanda | 17,436 | 751 | 0 | 0 | 18,187 |
| Sao Tome | 6,233 | 397 | 0 | 0 | 6,630 |
| Senegal | 18,864 | 2,232 | 0 | 0 | 21,096 |
| Sierra Leone | 20,011 | 0 | 0 | 0 | 20,011 |
| South Africa | 0 | 451 | 0 | 0 | 451 |
| South Sudan | 0 | 2,219 | 0 | 0 | 2,219 |
| Swaziland | 11,121 | 0 | 0 | 0 | 11,121 |
| Tanzania | 17,048 | 0 | 0 | 0 | 17,048 |
| The Gambia | 12,379 | 584 | 0 | 0 | 12,963 |
| Togo | 8,020 | 0 | 0 | 0 | 8,020 |
| Uganda | 7,287 | 6,784 | 0 | 0 | 14,071 |
| Zambia | 27,938 | 959 | 0 | 0 | 28,897 |
| Zimbabwe | 27,143 | 0 | 0 | 0 | 27,143 |
| Total | 549,127 | 28,466 | 30,009 | 54,377 | 661,979 |
